# Stability and Digestive Properties of a Dual-Protein Emulsion System Based on Soy Protein Isolate and Whey Protein Isolate

**DOI:** 10.3390/foods12112247

**Published:** 2023-06-02

**Authors:** Ting-Ting Gao, Jing-Xue Liu, Xin Gao, Guo-Qi Zhang, Xiao-Zhi Tang

**Affiliations:** 1 College of Food Engineering, Harbin University of Commerce, Harbin 150028, China; 2 College of Food Engineering, Jilin Agricultural Science and Technology University, Jilin 132101, China; 3 Collaborative Innovation Center for Modern Grain Circulation and Safety, Key Laboratory of Grains and Oils Quality Control and Processing, College of Food Science and Engineering, Nanjing University of Finance and Economics, Nanjing 210023, China

**Keywords:** soybean protein isolate, whey protein isolate, dual-protein emulsion, stability, digestive properties

## Abstract

The stability and digestive properties of a dual-protein emulsion consisting of soy protein isolate (SPI) and whey protein isolate (WPI) have been systematically studied. The results showed that the particle size and viscosity of the dual-protein emulsion system decreased continuously with the increase in WPI, and this might be related to the large amount of electric charge on the surface of the emulsion droplets. Dual-protein emulsions with ratios of 3:7 and 5:5 showed the highest emulsion activity, while emulsion stability increased with the increase in WPI. The thicker adsorption layer formed at the interface might have contributed to this phenomenon. After in-vitro-simulated digestion, the emulsion droplet particle size increased substantially due to the weakened electrostatic repulsion on the droplet surface, especially for the intestinal digestion phase. Meanwhile, WPI accelerated the release of free fatty acids in the digestion process, which played a positive role in the nutritional value of the dual-protein emulsion. In accelerated oxidation experiments, WPI also improved the antioxidant properties of the dual-protein emulsion system. This study will provide a new insight and necessary theoretical basis for the preparation of dual-protein emulsions.

## 1. Introduction

Protein-based colloidal systems were considered to play an important role in modern food processing [1]. Combining two or more proteins at different ratios was an effective strategy to provide sufficient essential amino acids at low protein doses. Dual-protein-based foods referred to edible protein sources, with two proteins as the major components, which not only achieved nutritional complementarity, but also met the dietary nutritional needs of consumers [2]. The combined application of plant and animal proteins has been shown to significantly enhance protein utilization [3]. Soybean protein isolate (SPI) and whey protein isolate (WPI), as typical representatives of plant and animal proteins, respectively, would provide ideal raw material sources for the construction of dual-protein systems [4]. The replacement of some animal proteins with plant proteins in formulae has been proposed to be beneficial in improving the sustainability of the products [5].

SPI was widely applied in food processing because of its low cost, high yield and high bioefficiency. It contained a variety of surface-active globulins (2S, 7S, 11S, 15S) that lowered the surface tension to promote emulsion formation [6]. Due to the good emulsification properties of soy protein isolate, researchers have developed a large number of emulsion-based food systems and evaluated their processing characteristics [6,7]. For example, a new W/O/W emulsion based on SPI has recently been designed [6]. The microscopic mechanisms by which soy protein hydrolysates and polysaccharides co-stabilize emulsions have also been thoroughly discussed recently [7]. In addition, WPI, consisting mainly of β-lactoglobulin, α-lactalbumin, bovine serum albumin and immunoglobulin, was widely used as a protein source in food processing due to its very high digestibility (85–90%) and high organismal metabolism rate [5,8]. The advantages offered by WPI not only for traditional emulsions, but also for other new colloidal food products were widely applied [8]. Its good effect in scavenging DPPH, ABTS and hydroxyl radicals made it a competitive protein-based additive [9].

Currently, research on dual-protein emulsions is flourishing. Oil-in-water emulsions composed of Cape hake and pea proteins were found to possess higher viscosity than single proteins [10], which would provide guidelines for the development of highly stable emulsion-based foods. It has also been suggested that the dual-protein system could fill the gap at the oil–water interface more efficiently and impart higher interfacial stability to the emulsion due to the presence of different structural domains of different proteins, while SPI and meat proteins might undergo competitive adsorption at the oil–water interface [11]. For composite protein emulsions, the protein ratio was the key to stability [12]. Proteins with fast adsorption rates might dominate the interface. Moreover, the molecular flexibility of proteins was also an important factor affecting competitive adsorption at the interface.

Consequently, we attempted to construct a dual-protein emulsion system using SPI and WPI, and further investigated the effect of the ratio of the two proteins on the stability and digestive properties. This research has developed a novel model emulsion system that will provide effective guidance for quality control of emulsion-based foods and the development of the food colloid interface theory.

## 2. Materials and Methods

### 2.1. Materials

SPI (purity > 90%) and WPI (purity > 90%) were purchased from Shanghai Yuanye Biotechnology Co., Ltd. (Yuanye Biotechnology Co., Ltd., Shanghai, China). All other reagents were provided by Sinopharm (Sinopharm Chemical Reagent Co., Ltd., Beijing, China).

### 2.2. Preparation of Dual-Protein Emulsions

Based on existing methods in our laboratory, the SPI and WPI were mixed in mass ratios of 1:9, 3:7, 5:5, 7:3 and 9:1. They were dissolved in deionized water and the total protein concentration was set to 10 mg/mL. 100 mL of the dual-protein solution and 20 mL of soybean oil were then mixed and homogenized at a high speed for 1 min at 8000 r/min (Ultra Turrax, Janke and Kunkel, Staufen, Germany). The pH was adjusted to 7.0. The resulting dual-protein emulsion was used for subsequent determinations.

### 2.3. Particle Size and Zeta Potential Determination

First, the dual-protein emulsion was diluted 800 times with a 10 mmol/L phosphate buffer (pH 7.0) [4]. A laser particle size meter (Malvern Instrument Co., Ltd., Malvern, UK) was used to determine the particle size and potential of the samples. The particle size and potential of the digested emulsions were also determined using the same method.

### 2.4. Viscosity Determination

The DHR-1 rotational rheometer (TA Instrument, New Castle, DE, USA) was used to determine the viscosity of a dual-protein emulsion. An appropriate amount of emulsion (1 mL) was placed on a sample platform with a plate diameter of 40 mm. The spacing between the plate and the platform was 1 mm, the shear rate was 1.0 s^−1^ and the temperature was 25 °C [10].

### 2.5. Determination of Emulsion Activity and Stability

A dual-protein solution at a concentration of 10 mg/mL was homogenized with continuously added soybean oil at 8000 r/min for 1 min. Emulsification activity and stability were calculated using the following equations [13]:

(1) $$EAI\left(m^{2}/g\right)=\frac{4.606\times A_{500}}{C\times \left(1-Ø\right)\times 10^{4}}\times dilution\;factor$$

where C is the protein concentration (g/mL) before emulsification, ø is the volume fraction (*v*/*v*) of the oil phase in the emulsion and A_500_ is the absorbance at 500 nm.

(2) $$ESI\left(m^{2}/g\right)=\left(A_{t}/A_{0}\right)\times 100$$

where A_t_ and A_0_ represent the absorbance at 60 min and 0 min, respectively.

### 2.6. Interfacial Adsorption Protein Determination

The prepared dual-protein emulsion was centrifuged at 12,000 r/min for 30 min until the emulsion was stratified. The lower clear night was carefully removed by syringe and filtered (0.45 μm). The protein concentration of the filtrate was determined by Lowry’s method. The interfacial protein content and adsorption rate were calculated using the following equations [14]:

(3) $$\Gamma \left(mg/m^{2}\right)=\left(C_{INI}-C_{SER}\right)\left(1-Ø\right)\times \frac{d}{6Ø}$$

(4) $$AP\left(\%\right)=\left(C_{INI}-C_{SER}\right)\times \frac{100}{C_{INI}}$$

where C_INI_ and C_SER_ are the protein concentration before emulsion formation and the protein concentration of the hypolimus, respectively, ø is the volume fraction of the oil phase and d is the average particle size of the surface area of the emulsion in the SDS solution (1%, *w*/*v*).

### 2.7. In-Vitro-Simulated Digestion

According to the in vitro simulation digestion model, the digestive environment of 2 parts of the stomach and small intestine were simulated to investigate the digestive properties of the dual-protein emulsion [15].

Of emulsion, 20 mL was added to 20 mL of the stomach simulant. The pH was adjusted to 2.5 and the mixture was stirred in a 37 °C water bath at 150 r/min for 1 h. The stomach simulant contained pepsin (3.2 mg/mL), NaCl (2 mg/mL) and HCl (0.7%, *v*/*v*) at a pH of 1.5.

The stomach digest (30 mL) was added to 3.5 mL of bile salt extract (5 mg/mL), 3.0 mL of salt solution (5 mmol/L CaCl_2_, 100 mmol/L NaCl) and 1.5 mL of trypsin solution (1.6 mg/mL). The pH of the mixed solution was adjusted to 7.0 and stirred at 150 r/min for 2 h in a water bath at 37 °C.

### 2.8. Determination of Free Fatty Acid Release

During the intestinal digestion phase, the free fatty acids were released and NaOH (0.25 mol/L) was used to neutralize the fatty acids and maintain the pH of the system at about 7.0. The consumption of NaOH was recorded throughout the process with an automatic titrator, which was used to calculate the free fatty acid release rate with the following equation [16]:

(5) $$FFA（\%）=100\times \left(\frac{V_{NaOH}\times m_{NaOH}\times M_{lipid}}{w_{lipid}\times 2}\right)$$

where V (L) is the volume of NaOH consumed, m (mol/L) is the molar concentration of NaOH, w (g) is the total mass of triglycerides and M is the average molecular mass of the triglycerides (876.56 g/mol for soybean oil).

### 2.9. Determination of Oxidative Stability

The dual-protein emulsion was subjected to accelerated oxidation in a thermostat at 40 °C. After 48 h, 0.2 mL of the sample was mixed into 5.0 mL of TBA reagent. The mixed system was fixed to 10 mL and further boiled in a water bath for 15 min. The cooled solution was filtered (450 nm) and the absorbance at 532 nm was recorded [17].

### 2.10. Data Statistics and Analysis

All tests were repeated at least three times and the data were expressed as the mean ± standard deviation (Duncan’s new multiple range test). SPSS 25 and Origin 2021 were used for data analysis and visualization. Significance analysis was performed at the level of *p* < 0.05. Different letters represented significant differences between the data.

## 3. Results and Discussion

### 3.1. Particle Size, Zeta Potential and Viscosity Analysis

**Figure 1:**
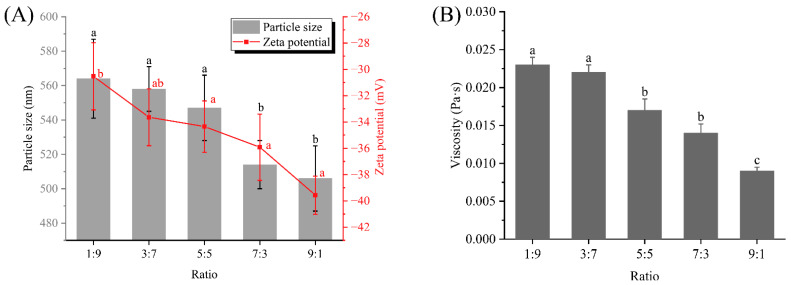
Particle size and zeta potential of emulsions prepared at different ratios (**A**). Viscosity of emulsions prepared at different ratios (**B**). Different letters represent significant differences between data.

The emulsion particle size was not only related to the protein loading at the interface, but also to the emulsification ability of the emulsifier itself adsorbed at the interface [6]. Figure 1A shows the emulsion droplet particle size and zeta potential of dual-protein emulsions prepared at different ratios. It was clear that the particle size decreased continuously from 564 nm at 1:9 to 506 nm at 9:1 as the content of WPI increased. In particular, after the predominance of WPI (7:3 and 9:1), the particle size decreased significantly (*p* < 0.05). In general, a smaller particle size predicted a more stable emulsion system. Therefore, this phenomenon suggested that WPI might have a positive effect on the stability of dual-protein emulsions, which might be due to its better interfacial affinity. The formation of thick film structures after more proteins were adsorbed at the interface would contribute to a significant decrease in interfacial tension, which helped the colloidal system reach thermodynamic stability [18]. In previous studies, researchers also found that the amount of interfacially adsorbed proteins was closely related to the particle size of the emulsion droplets [14]. Meanwhile, the net surface charge of the emulsion droplets increased with the increase in WPI. Different protein ratios could induce different interactions between the two proteins (e.g., hydrophobic interactions, hydrogen bonding interactions and van der Waals forces), and this difference further resulted in a protein conformational transition. The structurally rearranged protein surface was heavily charged and the resulting stronger electrostatic repulsion reduced the probability of collision and agglomeration between emulsion droplets, which led to a smaller particle size and higher emulsion stability. In addition, in emulsion systems, a smaller particle size was often accompanied by a higher surface charge, which predicted lower interfacial tension and better emulsion stability. Of course, in order to obtain smaller droplets, higher homogenization speeds could also be employed to prepare emulsions by strong shear action.

The apparent viscosities of the dual-protein emulsions at different ratios were given in Figure 1B. A ratio of 1:9 showed the highest viscosity, with a value of 0.023 Pa·s, and 9:1 showed the lowest viscosity, with a value of 0.009 Pa·s. Viscosity was not only related to the amount of protein and oil, but was also influenced by the dispersion state of the protein and oil, as well as the intermolecular interactions [19]. Since the total protein concentration and the amount of oil phase were consistent in different samples, this phenomenon could only be caused by differences in intermolecular interactions in the emulsion system. Theoretically, the hydration capacity of WPI was stronger than that of soy protein isolate [9], so the soy protein isolate adsorbed on the surface of emulsion droplets might flocculate and lead to an increase in viscosity. Moreover, due to the difference in interfacial affinity, soybean and WPIs adsorb from the aqueous phase to the oil–water interface in a competitive adsorption manner [20]. During competitive adsorption, differences in the kinetics of the two proteins in the diffusion, permeation and rearrangement phases all led to different interfacial protein compositions in different samples, and further affected the alignment state and macroscopic viscosity among the milk droplets. These emulsion droplets with different interfacial films might also interact differently with the aqueous phase under shear. Overall, the WPI-dominated dual-protein emulsions possessed superior stability.

### 3.2. Emulsification Activity and Stability Analysis

**Figure 2:**
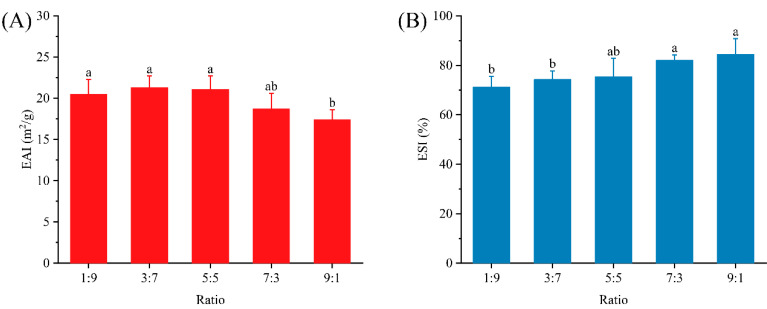
Emulsifying activity (**A**) and emulsion stability (**B**) of emulsions prepared at different ratios. Different letters represent significant differences between data.

As shown in Figure 2A, there was no significant difference (*p* > 0.05) in the emulsification activity of the samples with ratios of 1:9, 3:7 and 5:5. Subsequently, the emulsification activity showed a decreasing trend and reached the minimum value (17.4 m^2^/g) at 9:1. Emulsification activity was the area per unit mass of emulsifier that stabilizes the oil–water interface during the formation of emulsions between the aqueous and oil phases under homogeneous action. The increase in emulsification activity contributed to the formation of emulsions and it mainly characterized the protein–protein/lipid interactions [13]. Thus, the SPI-dominated system would stabilize more of the oil–water interface. This might be attributed to the fact that SPI could modulate the contact angle more efficiently, allowing for the better wetting of the oil–water interface. However, the emulsification activity of the samples with ratios of 3:7 and 5:5 showed a small increase compared to the samples with 1:9, indicating that the appropriate addition of WPI in a SPI-dominated system helped improve the interfacial activity of the dual-protein system. Indeed, this phenomenon also confirmed that the dual-protein system does have the potential to improve the emulsion processing characteristics. Nevertheless, although there was a small decrease in the emulsification activity of the whey-isolate-dominated system, it was maintained at a high level. Moreover, the smaller particle size (Figure 1A) also meant that the specific surface area of the emulsion droplets was larger, which was also beneficial for emulsion stability.

The emulsion stability of the dual-protein emulsion system was given by Figure 2B. Emulsion stability was the ability of the oil–water interface stabilized by the emulsifier to resist external changes. A larger value indicated that the aqueous and oil phases are less likely to undergo phase separation. It was observed that the emulsion stability of the 9:1 sample was improved by 18.68% compared to the sample with a ratio of 1:9, implying that the WPI significantly improved the stability of the dual-protein emulsion (*p* < 0.05). Figure 1A showed that the zeta potential of the WPI-dominated system was higher, and the strong repulsive effect made the droplets less prone to destabilizing processes such as agglomeration and flocculation due to gravity and Brownian motion. It was also possible that more WPIs were adsorbed at the interface. The thicker stacking layer and lower interfacial tension allowed for enhanced mechanical properties and resistance of the interfacial protein film structure. According to previous studies, this enhancement relied mainly on protein surface hydrophobicity, surface charge and adaptive change of molecular conformation at the interface [21]. The hydrophilic and hydrophobic domains of the protein molecular structure were embedded in the aqueous and oil phases, respectively, as a way to achieve interfacial stability. Research on the heat stability of milk proteins suggested that it would unfold at lower temperatures, which might favor the growth of interfacial films [22]. Additionally, molecular flexibility was critical for emulsion stability. The increase in WPI might also induce an improvement in the molecular flexibility of soy protein isolate by enhancing protein–protein interactions, which made the adsorbed protein less susceptible to desorption from the interface [23]. That is, the increase in WPI content was beneficial for the physical stability of the dual-protein emulsion system.

### 3.3. Interfacial Protein Adsorption Rate and Loading Analysis

**Figure 3:**
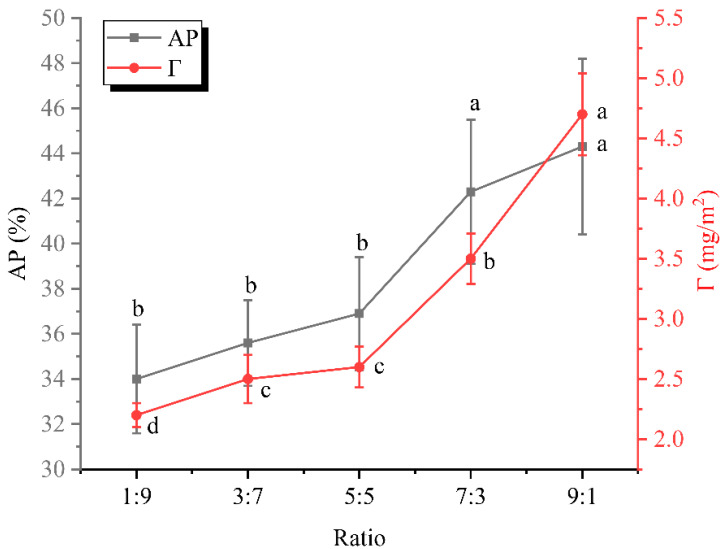
Protein adsorption percentage (AP) and interfacial protein loading (Γ) of emulsions prepared at different ratios. Different letters represent significant differences between data.

Figure 3 displays the protein adsorption rate and loading of the dual-protein system at the oil–water interface. Since the formation and stability of emulsions depended on the adsorption and rearrangement of proteins at the interface, the discussion of protein adsorption rates and loads was essential to understand the stability of the system. As shown in Figure 3, the interfacial protein adsorption percentage and load showed a simultaneous increasing trend, whereby for a given system, the more proteins adsorbed from the aqueous phase to the interface, the higher the load at the interface. The trend in Figure 3 indicated that the whey isolate promoted the interfacial migration behavior of the dual-protein system, and this might be due to the more stretchy molecular structure. In competitive adsorption, WPI might dominate due to its superior interfacial affinity. However, only a faster adsorption rate was still not enough; only long-time stabilization at the interface could resist the destabilization of the emulsion system [7]. Of course, since both soy protein isolate and WPI contained multiple subunits, subunit–subunit interactions might also lead to a stronger interfacial adsorption behavior [7]. Moreover, it was noticed that the emulsion stability in Figure 2B was enhanced with increasing WPI, and therefore, it could be speculated that WPI enhanced the emulsion stability by increasing the amount of interfacial adsorption. In fact, WPI was widely considered to be a good interfacial stabilizer. Other researchers have suggested that the more proteins accumulated at the oil–water interface, the more elastic characteristics of the interfacial membrane tended to be evident, meaning that the solid-like nature of the membrane depended heavily on the amount of protein (or certain subunits) adsorbed [14,18]. Alongside that, protein–protein interactions at the interface should not be neglected. Hence, appropriately increasing the proportion of WPI in the dual-protein emulsion system might be an effective strategy to obtain a higher stability.

### 3.4. Particle Size and Zeta Potential Analysis after In-Vitro-Simulated Digestion

**Figure 4:**
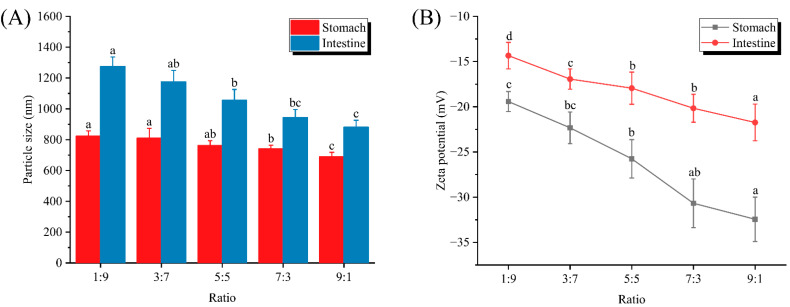
Particle size (**A**) and zeta potential (**B**) of emulsions prepared at different ratios after in-vitro-simulated digestion. Different letters represent significant differences between data.

The digestive properties of emulsions could be assessed by in-vitro-simulated digestion models, and we first discussed the digestive properties of different ratios of dual-protein emulsion systems in terms of particle size and surface charge at different digestion stages (stomach and intestine). As shown in Figure 4A, the particle size range of the dual-protein emulsion after stomach simulation was 689–823 nm, which was higher than the particle size range in Figure 1A. Moreover, the particle size after simulated digestion kept decreasing with the increase in WPI. On the one hand, the lower pH and higher ionic strength in the stomach simulant caused the aggregation of emulsion droplets. On the other hand, pepsin disrupted the interfacial membrane structure and thus led to the destabilization of the emulsion [15]. Further, the particle size of the dual-protein emulsion treated with the small intestine mimic was larger in the range of 881–1275 nm, which implied a complete disintegration of the emulsion system. Interfacial protein hydrolysis and desorption caused by trypsin and bile salts were the main reasons for this phenomenon [24]. After the triglycerides were broken down into glycerol and fatty acids, they easily came into contact with bile salts and formed micelles. Similarly, it was easily observed that the digested emulsion droplets maintained a smaller particle size with the increase in WPI, suggesting that WPI also conferred greater stability to the system during the simulated intestinal digestion. It should also be noted that an increase in particle size might also be evidence that the digestion products (free fatty acids, monoglycerides, free amino acids, peptides and phospholipids, etc.) were assembled at different sizes [25]. These digestion products could approach each other and agglomerate through electrostatic interactions or hydrophobic interactions and adversely affect the subsequent absorption process. In this sense, it seemed that WPIs would facilitate the release, absorption and utilization of nutrients.

Figure 4B illustrates the zeta potential of the emulsion droplets after simulated digestion. It could be observed that, similar to before digestion, the surface charges all increased with the increase in WPI, which was consistent with the results of particle size determination. However, the surface charge after digestion was significantly less than that of the undigested ones (Figure 1A), which precisely confirmed the fact that the particle size of the emulsion droplets increased after digestion. After the interfacial protein was disrupted by the digest, the net surface charge was drastically reduced and the weakened repulsive force was not sufficient to resist the agglomeration of the emulsion droplets, which led to the increase in particle size. Furthermore, the samples after simulated small intestine digestion showed the smallest zeta potential, which was not only consistent with the results of the particle size test, but also indicated that small intestine digestion would have a more significant effect on the stability of the dual-protein emulsion [8].

### 3.5. Free Fatty Acid Release Curve Analysis

**Figure 5:**
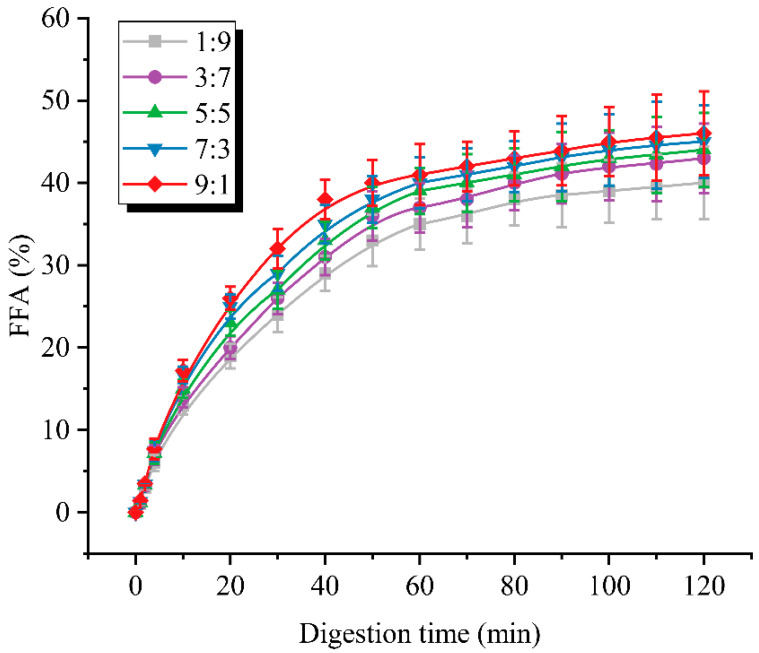
Free fatty acid release from emulsions prepared at different ratios during in-vitro-simulated digestion.

The release rate and amount of free fatty acids released were important indicators to evaluate the digestive characteristics of the emulsions. It could be seen in Figure 5 that each sample released free fatty acids at a very fast rate during the first 40 min, and then, the release rate gradually slowed down and showed a steady trend. The final free fatty acid release rates of the five samples were 40.0%, 43.1%, 44.2%, 45.0% and 46.4%, with the increase in the percentage of WPI, respectively. This phenomenon might be attributed to the easy formation of agglomerated structures of SPI at the oil–water interface, where the adsorption and contact of bile salts and trypsin were inhibited due to the spatial site barrier effect [13]. As a result, the rate and amount of free fatty acid release was lower for emulsions containing more SPI. According to previous studies [26], the hydrolysis of triglycerides at the interface consists of three key stages: (i) migration of bile-salt–lipase from the aqueous phase and binding at the interface; (ii) hydrolysis of triglycerides to monoglycerides and two free fatty acids; and (iii) desorption of the inhibited products by dissolving them in mixed micelles of bile salts and phospholipids. According to the above theory, bile salts played a key role in fat hydrolysis and free fatty acid release. Thus, the differences in the degree of lipid hydrolysis and the rate of free fatty acid release among samples with different ratios might be caused by the different affinities of interfacial protein films for bile salts. Furthermore, the small particle size (Figure 4A) often predicted a larger specific surface area, which also provided favorable conditions for adequate contact between the bile salts and the interface [21]. Overall, the rate and amount of free fatty acid release increased with the increase in WPI, implying that the digestive properties of the dual-protein emulsion system were enhanced, and its potential nutritional value was improved.

### 3.6. Oxidation Stability Analysis

**Figure 6:**
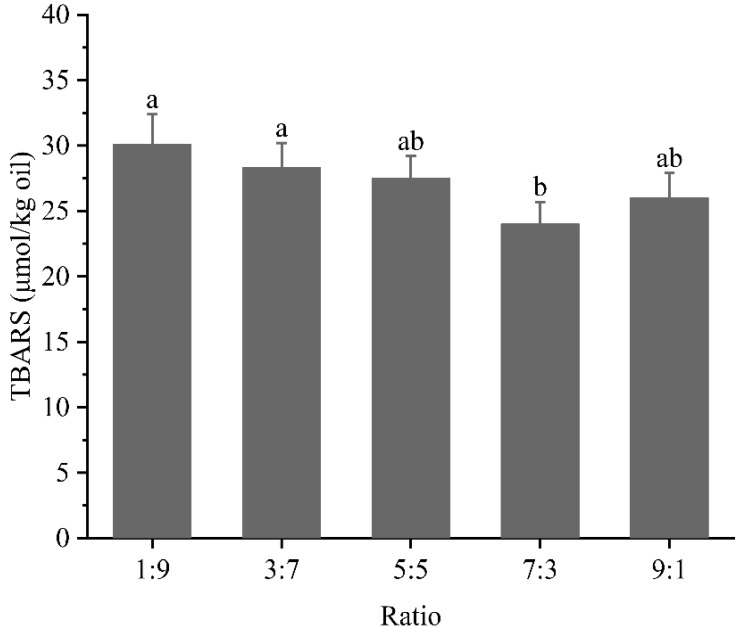
TBARS values of emulsions prepared at different ratios after oxidation. Different letters represent significant differences between data.

Figure 6 presented the TBARS values of the dual-protein emulsion system after oxidation, which was an essential indicator of the degree of lipid oxidation. Obviously, the dual-protein emulsion system exhibited the worst oxidative stability, with a TBARS value of 30.1 μmol/kg at a ratio of 1:9. As the percentage of WPI continued to increase, the emulsion showed superior resistance to oxidation. In particular, at a ratio of 7:3, the TBARS value was 24.0 μmol/kg. Compared to 1:9, the decrease in TBARS reached 20.27%, which was an improvement that should not be overlooked. A lower TBARS value meant that the dual-protein emulsion system could exhibit more stable physical properties and nutritional value during storage or processing [27]. In particular, the unsaturated fatty acids in the oil phase could be more protected. This phenomenon could perhaps be explained in two ways. On the one hand, Figure 2B indicates that more WPI could confer higher emulsion stability to the dual-protein emulsion system, which implies that the droplets were less prone to destabilization during storage. The oil phase was sufficiently encapsulated in the dual-protein film structure, thus blocking the direct contact of oxidants with the oil. On the other hand, the data in Figure 3 demonstrated a higher interfacial protein load for the WPI-dominated system. The large amounts of proteins adsorbed at the oil–water interface tended to form a thicker interfacial barrier, which not only facilitated resistance to emulsion destabilization, but also inhibited the attack of oxygen or metal ions, thus weakening the free radical chain oxidation reaction [28]. Similarly, previous reviews indicated that proteins could adsorb to the surface of emulsion droplets to form a physical barrier, thus limiting the oxidation process of polyunsaturated fatty acids [29]. Overall, the interfacial protein adsorption state might be a key factor affecting the oxidative stability of the dual-protein emulsion.

## 4. Conclusions

As WPI gradually dominated the dual-protein system, the surface charge of the emulsion droplets increased substantially and led to a decrease in emulsion particle size. This variation was considered necessary to improve the stability of the emulsion. The emulsification activity of the dual-protein emulsion was higher in the system dominated by soy protein isolate. Alongside that, WPI was favorable for emulsion stability, probably because WPI promoted more protein adsorption at the oil–water interface. After in-vitro-simulated digestion, the emulsion droplets became significantly larger, especially for the samples after intestinal digestion. During digestion, the rate and amount of free amino acids released from samples with different ratios differed significantly, mainly showing a dose dependence of WPI. Additionally, the dual-protein emulsion with a ratio of 3:7 showed the best antioxidant properties during the oxidation phase. These findings were important references for the development of new emulsion-based foods or improving the quality of existing products such as salad dressings, mayonnaise, dairy beverages, etc. How the stability and digestive properties of dual-protein emulsions will change under the regulation of different environmental factors is a major focus of subsequent research.

## Data Availability

The data presented in this study are available on request from the corresponding author.

## References

[1] Tarahi M., Ahmed J. (2023). Recent advances in legume protein-based colloidal systems. Legume Sci..

[2] Zhou X., Zhang C., Zhao L., Zhou X., Cao W., Zhou C. (2022). Effect of pre-emulsion of pea-grass carp co-precipitation dual protein on the gel quality of fish sausage. Foods.

[3] Jie Y., Chen F. (2022). Progress in the application of food-grade emulsions. Foods.

[4] Zhang X., Zhang S., Xie F., Han L., Li L., Jiang L., Qi B., Li Y. (2021). Soy/whey protein isolates: Interfacial properties and effects on the stability of oil-in-water emulsions. J. Sci. Food Agric..

[5] Alves A., Martha L., Casanova F., Tavares G. (2022). Structural and foaming properties of whey and soy protein isolates in mixed systems before and after heat treatment. Food Sci. Technol. Int..

[6] Liu J., Zhou H., Tan Y., Muriel Mundo J.L., McClements D.J. (2021). Comparison of plant-based emulsifier performance in water-in-oil-in-water emulsions: Soy protein isolate, pectin and gum Arabic. J. Food Eng..

[7] Ding Y., Chen L., Shi Y., Akhtar M., Chen J., Ettelaie R. (2021). Emulsifying and emulsion stabilizing properties of soy protein hydrolysates, covalently bonded to polysaccharides: The impact of enzyme choice and the degree of hydrolysis. Food Hydrocoll..

[8] Cheng Y., Ofori Donkor P., Yeboah G.B., Ayim I., Wu J., Ma H. (2021). Modulating the in vitro digestion of heat-set whey protein emulsion gels via gelling properties modification with sequential ultrasound pretreatment. LWT.

[9] Hu M., McClements D.J., Decker E.A. (2003). Lipid Oxidation in Corn Oil-in-Water Emulsions Stabilized by Casein, Whey Protein Isolate, and Soy Protein Isolate. J. Agric. Food Chem..

[10] Tomé A.S., Pires C., Batista I., Sousa I., Raymundo A. (2015). Protein gels and emulsions from mixtures of Cape hake and pea proteins. J. Sci. Food Agric..

[11] Santiago L., Carrara C., González R. (2005). Interaction of Soy Protein Isolate and Meat Protein in a Model Emulsion System. Effect of Emulsification Order and Characteristics of Soy Isolate Used. Food Sci. Technol. Int..

[12] Wang T., Li F., Zhang H., Feng W., Wang R. (2022). Plant-based high internal phase emulsions stabilized by dual protein nanostructures with heat and freeze–thaw tolerance. Food Chem..

[13] Li Y., Zhong M., Xie F., Sun Y., Zhang S., Qi B. (2020). The effect of pH on the stabilization and digestive characteristics of soybean lipophilic protein oil-in-water emulsions with hypromellose. Food Chem..

[14] Liang H., Tang C. (2014). Pea protein exhibits a novel Pickering stabilization for oil-in-water emulsions at pH 3.0. LWT.

[15] Marefati A., Bertrand M., Sjöö M., Dejmek P., Rayner M. (2017). Storage and digestion stability of encapsulated curcumin in emulsions based on starch granule Pickering stabilization. Food Hydrocoll..

[16] Liu N., Li N., Faiza M., Li D., Yao X., Zhao M. (2021). Stability and in vitro digestion of high purity diacylglycerol oil-in-water emulsions. LWT.

[17] Keramat M., Ehsandoost E., Golmakani M.-T. (2023). Recent trends in improving the oxidative stability of oil-based food products by inhibiting oxidation at the interfacial region. Foods.

[18] Taha A., Ahmed E., Hu T., Xu X., Pan S., Hu H. (2019). Effects of different ionic strengths on the physicochemical properties of plant and animal proteins-stabilized emulsions fabricated using ultrasound emulsification. Ultrason. Sonochem..

[19] Zhang X., Qi B., Xie F., Hu M., Sun Y., Han L., Li L., Zhang S., Li Y. (2021). Emulsion stability and dilatational rheological properties of soy/whey protein isolate complexes at the oil-water interface: Influence of pH. Food Hydrocoll..

[20] Ho K.K.H.Y., Schroën K., San Martín-González M.F., Berton-Carabin C.C. (2018). Synergistic and antagonistic effects of plant and dairy protein blends on the physicochemical stability of lycopene-loaded emulsions. Food Hydrocoll..

[21] Ahn N., Imm J.-Y. (2023). Effect of phospholipid matrix on emulsion stability, microstructure, proteolysis, and in vitro digestibility in model infant formula emulsion. Food Res. Int..

[22] Euston S., Al-Bakkush A., Campbell L. (2009). Comparing the heat stability of soya protein and milk whey protein emulsions. Food Hydrocoll..

[23] Zhai J., Wooster T.J., Hoffmann S.V., Lee T.-H., Augustin M.A., Aguilar M.-I. (2011). Structural Rearrangement of β-Lactoglobulin at Different Oil–Water Interfaces and Its Effect on Emulsion Stability. Langmuir.

[24] Xiao J., Lu X., Huang Q. (2017). Double emulsion derived from kafirin nanoparticles stabilized Pickering emulsion: Fabrication, microstructure, stability and in vitro digestion profile. Food Hydrocoll..

[25] Li Q., He S., Xu W., Peng F., Gu C., Wang R., Ma Y. (2018). Formation, Stability and In Vitro Digestion of β-carotene in Oil-in-Water Milk Fat Globule Membrane Protein Emulsions. Food Biophys..

[26] Golding M., Wooster T.J. (2010). The influence of emulsion structure and stability on lipid digestion. Curr. Opin. Colloid Interface Sci..

[27] Feng J., Schroën K., Fogliano V., Berton-Carabin C. (2021). Antioxidant potential of non-modified and glycated soy proteins in the continuous phase of oil-in-water emulsions. Food Hydrocoll..

[28] Faraji H., McClements D.J., Decker E.A. (2004). Role of Continuous Phase Protein on the Oxidative Stability of Fish Oil-in-Water Emulsions. J. Agric. Food Chem..

[29] Kiokias S., Gordon M., Oreopoulou V. (2017). Effects of composition and processing variables on the oxidative stability of protein-based and oil-in-water food emulsions. Crit. Rev. Food Sci. Nutr..

